# Effect of nicotine on the energy metabolism of substantia nigra cells in MPTP-induced Parkinson’s disease

**DOI:** 10.22038/ijbms.2025.84754.18338

**Published:** 2025

**Authors:** Nikoloz Zhgenti, Otar Bibilashvili, Mariam Shengelia, George Burjanadze, Marine Koshoridze, Elene Davitashvili, Nana Koshoridze

**Affiliations:** 1 Department of Biology, Faculty of Exact and Natural Sciences, Iv. Javakhishvili Tbilisi State University, Tbilisi, Georgia; 2 Faculty of Medicine, Iv. Javakhishvili Tbilisi State University, Tbilisi, Georgia

**Keywords:** Energy metabolism, Mitochondrial permeability transition pore, Neurodegenerative diseases, Neuroprotective agents1, Nicotine, Parkinson’s disease

## Abstract

**Objective(s):**

Parkinson’s disease (PD) is a progressive neurodegenerative disorder affecting millions globally, with no current cure despite extensive research efforts. The neurotoxin MPTP is commonly used as a PD model by inhibiting mitochondrial complex I. Nicotine, the primary alkaloid in tobacco, has shown potential neuroprotective effects against neurodegenerative diseases, including PD, although the precise mechanisms remain unclear. This study aims to investigate the effects of nicotine on the energetic metabolism of substantia nigra cells affected by MPTP.

**Materials and Methods:**

We examined the impact of nicotine on glycolytic, Krebs cycle, and respiratory chain enzymes in substantia nigra cells, as well as mitochondrial and cytosolic creatine kinase activities. ATP levels, mitochondrial permeability transition pore (mPTP) activity, and PI3K-AKT-mTOR signaling pathway were also assessed. The study was performed on a mouse model where PD was induced by MPTP, followed by nicotine treatment.

**Results:**

Nicotine administration led to improvements in mitochondrial function, with enhanced ATP production, creatine kinase activity, and overall energetic metabolism. Nicotine corrected the energetic deficiencies induced by MPTP, likely through the activation of the PI3K-AKT-mTOR pathway, which is suppressed by MPTP.

**Conclusion:**

Our findings suggest that nicotine may exert neuroprotective effects in Parkinson’s disease by improving mitochondrial function and enhancing energetic metabolism, potentially via activation of the PI3K-AKT-mTOR pathway. This highlights nicotine’s potential as a therapeutic agent in mitigating PD-induced metabolic disturbances.

## Introduction

Parkinson’s disease (PD) is a progressive neurodegenerative disorder that ranks second in prevalence only after Alzheimer’s disease. Over the past 25 years, the global prevalence of PD has doubled. In 2019, it was estimated that more than 8.5 million people worldwide were living with PD (1, 2). According to some authors, it is projected that by 2040, PD will affect around 12 million people (3). The disease is characterized by the death of neurons in the substantia nigra (SN), leading to a range of neurological symptoms, including rigidity, resting tremors, and bradykinesia (4). The molecular basis of PD is inextricably linked to several key processes: the abnormal aggregation of alpha-synuclein, culminating in the formation of Lewy bodies; dysregulation of the ubiquitination process; and the emergence of oxidative stress as a consequence of mitochondrial dysfunction, which leads to the accumulation of pathological alpha-synuclein and the generation of reactive oxygen species (ROS) and reactive nitrogen species (RNS). Collectively, these factors contribute significantly to the degeneration and ultimate demise of dopaminergic neurons in the SN (5, 6). Among the many animal models of PD, the 1-methyl-4-phenyl-1,2,3,6-tetrahydropyridine (MPTP)-induced model is widely recognized for its ability to reproduce the selective degeneration of SN dopaminergic neurons, thereby providing valuable insights into the mechanisms of PD pathology. Furthermore, MPTP-induced neurotoxicity is associated with mitochondrial complex I inhibition, leading to oxidative stress, energy deficits, and dopaminergic cell death (7, 8).

Nicotine, an alkaloid found primarily in tobacco, has garnered significant attention for its neuroprotective properties. Several epidemiological studies suggest an inverse correlation between smoking and the incidence of PD, proposing that nicotine may exert protective effects on dopaminergic neurons (9–11). It has been shown that nicotine reduces oxidative stress caused by MPTP, enhances the activity of anti-oxidant enzymes, and lowers levels of reactive oxygen species (ROS) and reactive nitrogen species (RNS) (12). Nicotine has also been shown to affect various mitochondrial processes, including electron transport chain complex activity, uncoupling protein 1 (UCP1) expression, and the regulation of fission- and fusion-related proteins (13–15).

It also exhibits therapeutic effects in animal models of PD by reducing dopaminergic degeneration in the substantia nigra (SN) (16–17), increasing dopamine levels in the striatum (18–19), and influencing mitochondrial respiration (20). However, its impact on energy metabolism processes such as glycolysis, the citric acid cycle, creatine kinase activity, and the functional state of the mitochondrial permeability transition pore (mPTP) in Parkinsonian conditions remains unclear (21).

The energy metabolism of neurons, including those in the substantia nigra, is highly dependent on the proper functioning of mitochondria. The first step of neuronal energy metabolism is glycolysis, which occurs in the cytoplasm, where one glucose molecule is broken down into two molecules of pyruvate. This process generates a small amount of energy in the form of 2 ATP and 2 NADH molecules. The pyruvate produced in glycolysis is then transported into the mitochondria, where it is converted into acetyl-CoA by the enzyme pyruvate dehydrogenase. Acetyl-CoA enters the Krebs cycle, which takes place in the mitochondrial matrix. The Krebs cycle completes the oxidation of acetyl-CoA, producing CO₂, NADH, and FADH₂. The NADH and FADH₂ generated in both glycolysis and the Krebs cycle are then used in the electron transport chain (ETC).

The ETC, located in the inner mitochondrial membrane, uses the high-energy electrons from NADH and FADH₂ to power the movement of protons (H⁺) across the membrane, creating an electrochemical gradient (proton gradient). The flow of electrons through protein complexes in the ETC ultimately leads to the reduction of oxygen to water. This proton gradient drives ATP synthesis as protons flow back into the mitochondrial matrix through the enzyme ATP synthase, which phosphorylates ADP to form ATP. This process, known as oxidative phosphorylation, produces the bulk of ATP generated during cellular respiration. Together, glycolysis, the Krebs cycle, and the electron transport chain provide a continuous flow of energy, converting glucose into ATP in a coordinated, stepwise manner (22–24). The resulting ATP passes from the mitochondria to the cytosol, where it is used in various processes. However, part of the ATP in the mitochondria is oxidized to ADP by the mitochondrial isoform of the enzyme creatine kinase, which transfers the phosphate group from ATP to creatine, resulting in the high-energy compound phosphocreatine, which then passes into the cytosol, where it is consumed by the body when energy demand increases. The regeneration of ATP from phosphocreatine is catalyzed by the cytoplasmic isoform of creatine kinase. This process helps store energy in the form of phosphocreatine, which can be rapidly mobilized when needed (25).

**Figure 1:**
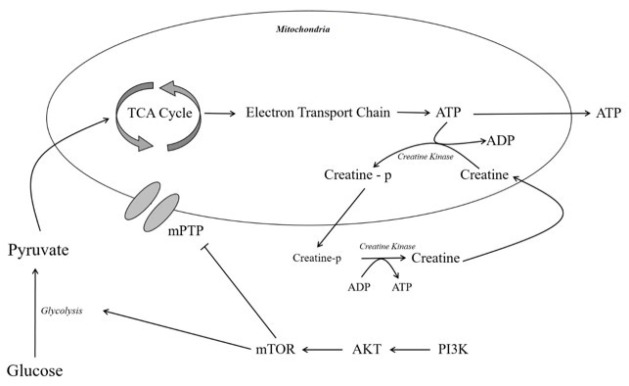
Schematic representation of the interconnections between respiratory metabolism, creatine kinase, the mitochondrial permeability transition pore (mPTP), and the PI3K-AKT-mTOR signaling pathway

It is well established that normal cellular energy metabolism is essential for the growth and development of both individual cells and the entire organism. This process is tightly regulated and influenced by a variety of factors, among which intracellular signaling pathways play a key role. Of particular importance is the phosphoinositide 3-kinase (PI3K)/protein kinase B (AKT)/mechanistic target of rapamycin (mTOR) signaling pathway, which warrants special attention due to its significant role in metabolic regulation. The PI3K/AKT/mTOR pathway is crucial for regulating cellular growth, survival, and metabolism. Upon activation, PI3K phosphorylates and activates AKT, facilitating its localization to the plasma membrane. AKT then initiates a number of downstream effects, one of the most important being the activation of mTOR (26). PI3K/AKT/mTOR signaling interfaces with key metabolic processes, particularly through the regulation of glucose uptake and glycolysis. Notably, hypoxia-inducible factor 1 alpha (HIF1α), a downstream effector of mTORC1, plays an important role in upregulating the expression of hexokinase 2 and pyruvate kinase M2, two critical, rate-limiting enzymes in the glycolytic pathway. Another downstream mechanism of mTORC2 involves the transcription factor c-MYC, which promotes the expression of heterogeneous nuclear ribonucleoproteins (hnRNPs) involved in the transcription of pyruvate kinase M2 (27–28). In addition to its metabolic roles, this pathway contributes to cellular protection against oxidative stress, in part by regulating the opening of the mitochondrial permeability transition pore (mPTP). Dysregulation of mPTP can lead to mitochondrial swelling and cell death, linking energy metabolism to cell survival pathways (29). Figure 1 illustrates the integration of these processes into a unified model, depicting cellular energy metabolism as a cohesive and dynamically regulated system.

This study aims to explore the effects of chronic nicotine administration on the energetic metabolism of substantia nigra cells in an MPTP-induced PD model. By examining the mitochondrial permeability transition pore functional state, the ATP production process, and the PI3K-AKT-mTOR signaling pathway, we aim to elucidate the potential protective mechanisms of nicotine in modulating the metabolic disturbances associated with PD. The findings from this research could provide valuable insights into novel therapeutic approaches targeting metabolic pathways in PD treatment.

## Materials and Methods

### Study design

**Figure 2:**
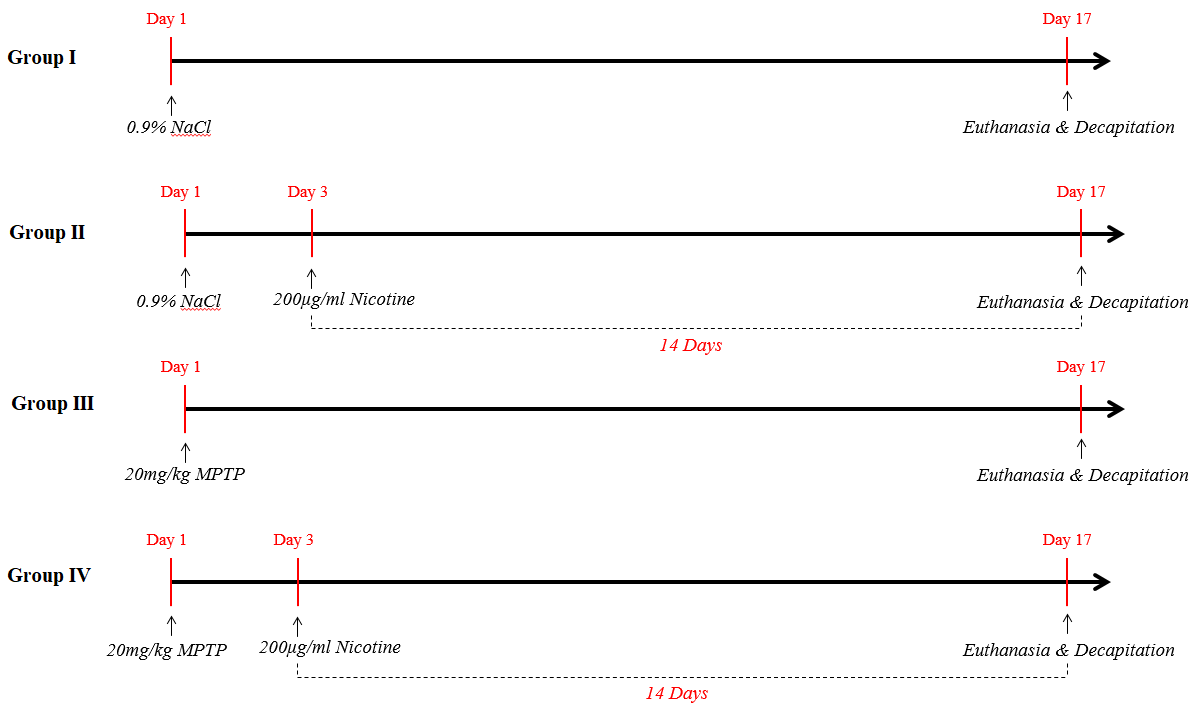
Study design

The research was conducted on BALB/c line laboratory mice, aged 8 weeks and weighing 22 ± 2 g. The animals were housed, cared for, and tested in accordance with the Guide for the Care and Use of Laboratory Animals, 2021 (30) at the Department of Biology, Iv. Javakhishvili Tbilisi State University and its vivarium. Throughout the study, the mice had unrestricted access to both water and standard laboratory-grade food. The ambient room temperature was rigorously maintained at 22 ± 2 °C to ensure a stable and comfortable environment for the animals. Furthermore, the mice were subjected to a controlled 12-hr light-dark cycle to mimic their natural circadian rhythm. Before the commencement of any experimental procedures, the mice were allowed a period of 7 days for acclimatization to the vivarium conditions. Following this adaptation phase, they were systematically partitioned into four distinct experimental groups, each composed of 40 mice (Figure 2): 

• Group I (G1) (control group): Mice received four intradermal injections of sterile physiological solution (0.9% NaCl) at 2-hr intervals, with a volume of 10 ml/kg. 

• Group II (G2): Mice were treated with drinking water containing nicotine bitartrate at a concentration of 10mg/kg, *ad libitum,* for 14 days. 

• Group III (G3): Mice received four intradermal injections of MPTP at 2-hr intervals, at a dose of 20 mg/kg, with a volume of 10 ml/kg. 

• Group IV (G4): Mice were injected intradermally with MPTP following the same protocol as Group G3, and 72 hours after the injection, they were given an aqueous nicotine solution for 14 days, similar to Group G2. 

The dosage of both the neurotoxin and nicotine was determined based on existing literature data (31-32). A fresh nicotine solution was prepared daily. According to the literature, the average experimental dose of transdermal nicotine is around 20 mg/kg, with a bioavailability of approximately 75–80% (33). In contrast, only about 30–40% of orally administered nicotine enters systemic circulation (34). Therefore, the oral dose used in this study (10 mg/kg) is below the typical transdermal equivalent. On the seventeenth day of the study, the animals from each group were euthanized in accordance with international guidelines (35). After euthanizing the animals, we decapitated them. (Figure 2) Then, their brains were removed and frozen at -80 °C. Using the rodent brain coronal matrice (RBMS-200C), stereomicroscope (Laxco LMS-Z230PMZS33), and mouse brain atlas (36), we dissected the substantia nigra. 

### Subcellular fractionation

Fractionation of brain tissue was performed as per Whittaker (1969). Briefly, mouse brains were homogenized in 0.32 M sucrose and centrifuged at 1,000× g for 10 min. The supernatant (S1) was collected and centrifuged at 17,000× g for 55 min, resulting in the S2 supernatant and the P2 pellet (crude mitochondrial fraction). S2 was centrifuged at 100,000× g for one hour to generate the supernatant (cytosol fraction). P2 was resuspended in 0.32 M sucrose and layered onto a 1.2 M, 0.8 M block sucrose gradient. Following centrifugation at 53,000× g for two hours, fractions A (0.32/0.8M boundary), B (0.8/1.2 M boundary), and C (pellet below 1.2 M) (pure mitochondrial fraction) were collected and centrifuged at 100,000× g for one hour to isolate membranes. Pure mitochondrial and cytosol fractions were used for further experiments (37).

### Determination of energetical metabolism enzyme activity

To study the glycolysis process, we measured the activity of several key enzymes involved in this pathway. First, we assessed the activity of hexokinase, the primary enzyme of glycolysis that catalyzes the phosphorylation of glucose. For this, we used a commercially available kit from MyBioSource (MBS9719202). In addition, we measured the activity of fructose-bisphosphate aldolase, another critical enzyme that catalyzes the reversible reaction splitting fructose 1,6-bisphosphate into two triose phosphates: dihydroxyacetone phosphate (DHAP) and glyceraldehyde 3-phosphate (G3P). This was done using the Fructose-bisphosphate Aldolase Test Kit (MBS265365).

Given the importance of pyruvate dehydrogenase as the key enzyme linking glycolysis to the tricarboxylic acid (TCA) cycle, we also measured its activity using the Pyruvate Dehydrogenase Test Kit (MBS8243249). To investigate the TCA cycle, we measured the activity of several critical enzymes, including aconitase (MBS8309682), alpha-ketoglutarate dehydrogenase (MBS8309683), Succinate Dehydrogenase (MBS8243220), fumarase (MBS7218132), and malate dehydrogenase (MBS8309689), using their respective enzyme activity kits. These measurements provided comprehensive insights into the functionality of glycolysis and the TCA cycle. 

In addition to these enzymes, the key enzyme creatine kinase, which plays an active role in cellular energy metabolism, was also analyzed. The activities of both mitochondrial and cytoplasmic isoforms of creatine kinase were measured using a test kit from Abcam (Ab155901).

### Determination of electron transport chain enzyme activity

To investigate ATP synthesis in detail, we analyzed the activity of the entire respiratory chain by measuring the enzymatic activities of its key complexes. This included Complex I (NADH: ubiquinone oxidoreductase), assessed using the Mybiosource kit (MBS8806971); Complex II (succinate dehydrogenase), measured with the kit (MBS8243220); Complex III (cytochrome bc1 complex), using the Complex III Test Kit (MBS3805803); and Complex IV (cytochrome c oxidase), evaluated with the kit (MBS037447). Additionally, we measured the activity of Complex V, also known as ATP synthase, using the ATP Synthase Activity Test Kit (MBS8305380). 

### Determination of ATP levels

To quantify ATP levels, we employed the Luminescent ATP Detection Assay Kit from Abcam (Ab113849). 

### Mitochondrial permeability pore (mPTP) opening

To determine the mitochondrial permeability pore (mPTP) opening, 2 ml mitochondrial suspension (approximately 0.5 mg protein) was added to 1 ml of an incubation medium prepared using 25 mM Tris-HCl buffer, containing 120 mM KCl, 3 mM KH₂PO₄, and 5 mM sodium succinate, adjusted to pH 7.4. To induce pore opening, CaCl₂ was added to a final concentration of 420 µM, while cyclosporin A, at a concentration of 0.42 µM, was used to inhibit this effect. Both CaCl₂ and cyclosporin A were introduced into the reaction mixture either sequentially or in equal volumes. Absorbance values ​​were measured spectrophotometrically at 520 nm, and pore opening was assessed based on changes in absorbance over a 30-minute period (38). After a stable initial optical density (OD) was obtained, the absorbance change was set to zero, and subsequent changes were recorded. Upon the addition of calcium chloride, the absorbance change indicated pore opening, as reflected by a decrease in OD, while cyclosporin A caused an increase in OD, reflecting pore closure. 

To assess mitochondrial swelling, a swelling buffer containing 70 mM sucrose, 230 mM mannitol, 3 mM HEPES, 2 mM Tris-base, 5 mM sodium succinate, and 1 μM rotenone was added to the mitochondrial suspension. The absorbance was measured spectrophotometrically at 540 nm. A decrease in optical density (OD) is proportional to mitochondrial swelling (39).

### PI3K-AKT-mTOR pathway Western blotting analysis

Substantia nigra cells from experimental animals were analyzed using Western blotting, following the standard method (40). The following primary antibodies were used: PI3 Kinase p85 Antibody (Cell Signaling #4292); Phospho-Akt (Ser473) Antibody (Cell Signaling #9271), and Phospho-mTOR (Ser2448) Antibody (Cell Signaling #2971). Immunoreactivity was detected by enhanced chemiluminescence autoradiography (ECL kit, Santa-Cruz Biotechnology). Protein concentrations were determined using a BCA protein assay kit (Thermo Scientific). Signal quantification from Western blots was assisted by ImageJ software.

### Chemicals and reagents

All the reagents were purchased from Sigma–Aldrich (Sigma–Aldrich Inc., St. Louis, USA) unless otherwise specified.

### Statistical analysis

Data are presented as mean ± standard error of the mean (SEM), and statistical analysis was performed using GraphPad Prism 9.0 software (GraphPad Prism 9.0, USA). To assess the assumption of normality, the Shapiro-Wilk test was applied. Only data that met the normality assumption were subjected to further analysis using a one-way ANOVA to determine statistical significance, followed by Tukey’s *post hoc* test for multiple comparisons. *P*-values of 0.05 or less were considered statistically significant.

## Results

### Energy metabolism enzyme activity

**Figure 3:**
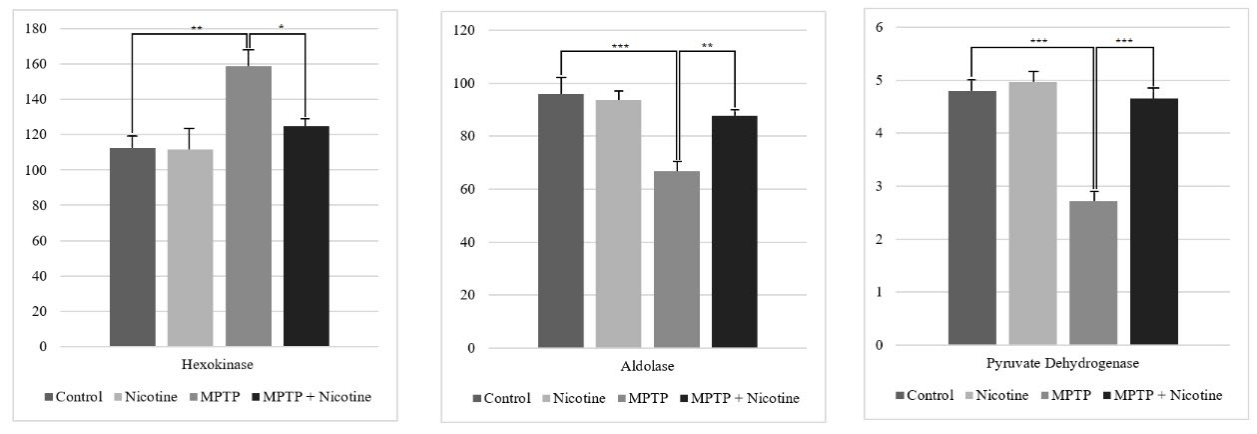
Activity of hexokinase, aldolase and pyruvate dehydrogenase (U/mg)

In the initial phase of the experiment, the activities of glycolytic enzymes in the substantia nigra cells of MPTP-treated laboratory mice were analyzed. These results were compared to data obtained after a 14-day nicotine treatment. Hexokinase activity increased by approximately 40% following MPTP administration (*P*≤0.001) but returned to normal levels in the MPTP + Nicotine group (*P*≤0.01). In contrast, aldolase activity decreased by about 30% after MPTP treatment compared to the control (*P*≤0.0001) but similarly normalized following nicotine administration (*P*≤0.001). Additionally, pyruvate dehydrogenase activity showed a 43% decrease post-MPTP treatment (*P*≤0.0001), with levels returning to baseline after chronic nicotine administration (*P*≤0.0001) (Figure 3).

**Figure 4:**
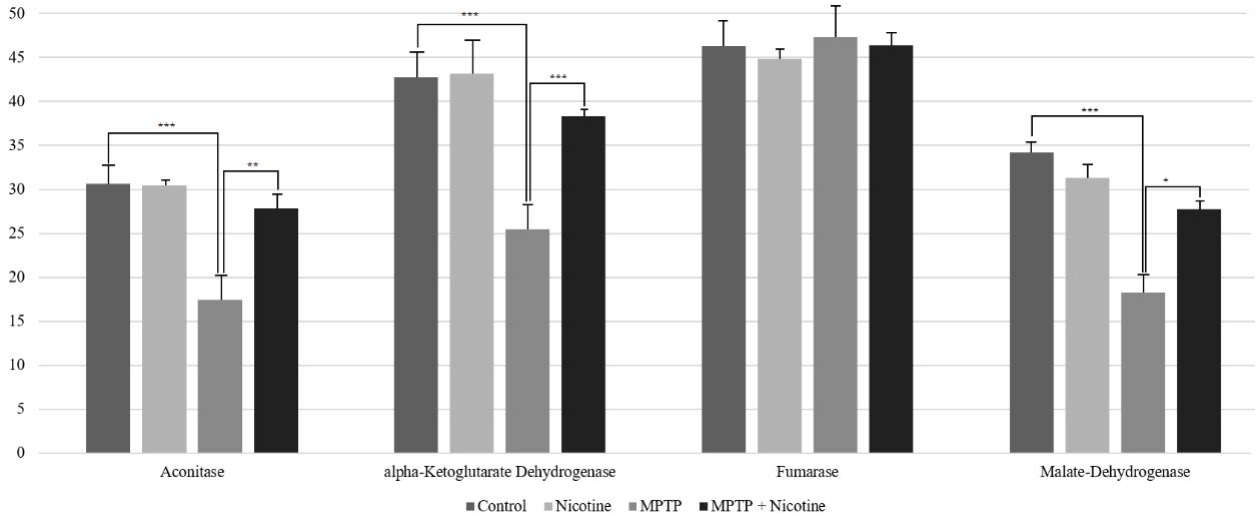
Activity of aconitase, alpha-ketoglutarate dehydrogenase, fumarase and malate- dehydrogenase (U/mg)

Regarding TCA cycle enzymes, most exhibited decreased activity in Group 3 (MPTP-treated) compared to the control group (*P*≤0.0001). However, these activities returned to normal levels following nicotine treatment (Group 4). For example, aconitase, alpha-ketoglutarate dehydrogenase, and malate dehydrogenase activities decreased by 40–45% in Group 3 but increased by approximately 50–60% after nicotine administration. In contrast, fumarase activity showed no significant changes (Figure 4). Notably, in healthy animals, nicotine administration (Group 2) did not affect the activities of glycolytic or TCA cycle enzymes. 

**Figure 5:**
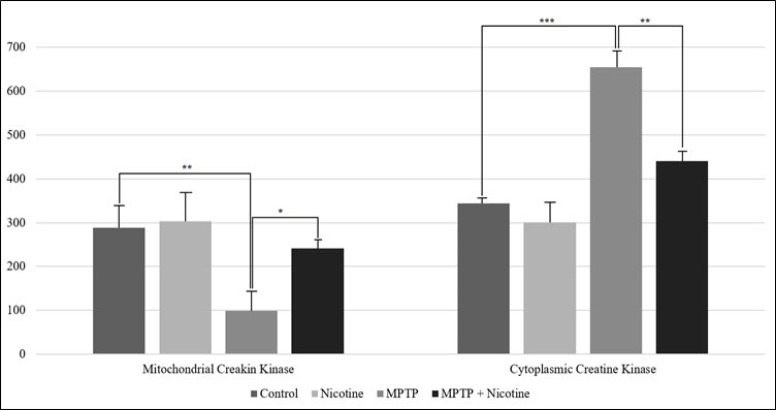
Ativity of mitochondrial and cytoplasmic isoforms of creatine kinase (mU/mg)

In addition to glycolytic and TCA cycle enzymes, the activity of creatine kinase, an enzyme involved in energy metabolism, was assessed in both its cytosolic and mitochondrial isoforms. The results are presented in Figure 5. MPTP exposure led to a nearly threefold decrease in mitochondrial creatine kinase activity (*P*≤0.001), while cytoplasmic isoform activity increased by approximately 90% (*P*≤0.0001). Following nicotine administration, the activity of both isoforms returned to normal levels (*P*≤0.01, *P*≤0.001) (Figure 5).

### Determination of electron transport chain enzyme activity

**Figure 6:**
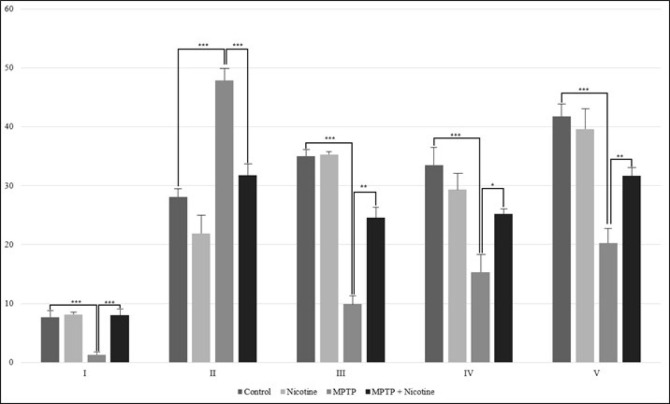
Activity of electrone transport chain enzyme complexes: I complex (NADH depended dehydrogenase); II complex (succinate dehydrogenase); III complex (cytochrome c reductase); IV complex (cytochrome c oxidase), and V complex (ATP synthase) (U/mg)

Based on the obtained data, it became relevant to investigate the direct effect of nicotine on ATP synthesis, specifically by examining the activity of mitochondrial respiratory chain complexes in the substantia nigra mitochondria of both control and MPTP-treated mice. As shown in Figure 6, the activity of all enzyme complexes in the respiratory chain significantly decreased by 50-80% (*P*≤0.0001), except for Complex II, whose activity increased by more than 70% (*P*≤0.0001). Following nicotine administration, the function of the electron transport chain approached normal levels. The activity of Complex II decreased, while the activities of all other complexes increased.

### Determination of ATP levels

**Figure 7:**
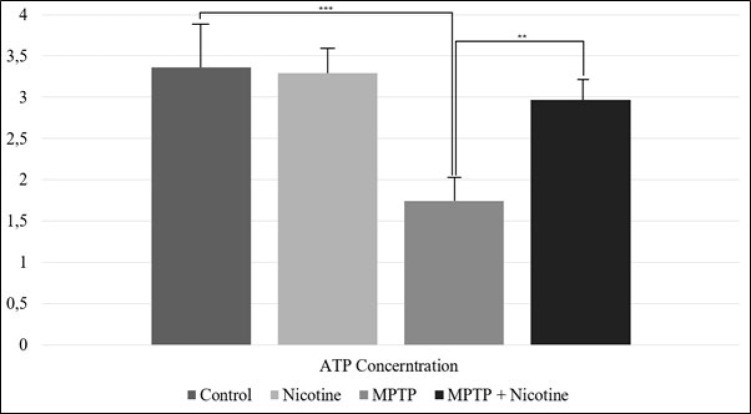
Concentration of ATP in SN cells (μM/mg)

The obtained results are consistent with the subsequent experiment, where the amount of ATP in the study cells was measured directly. Analysis of ATP levels revealed a sharp reduction in its concentration in the cells of the substantia nigra following MPTP exposure (*P*≤0.0001). However, nicotine administration significantly increased ATP levels, bringing them closer to normal (*P*≤0.001) (Figure 7).

**Figure 8:**
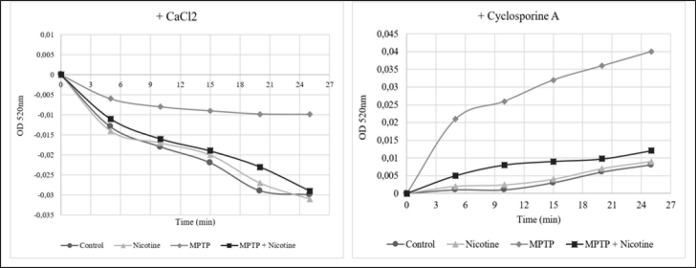
Activity of mPTP in substantia nigra cells (Change of optical density (OD) at 520 nm after CaCl_2_ (A), and cyclosporine A (B) addition

The obtained data provided insights into the functional state of mitochondria across the experimental groups. Specifically, we assessed the state of the mitochondrial permeability transition pore (mPTP), a key driver of changes in mitochondrial function. Analysis of mPTP activity revealed that all experimental groups, except the MPTP group, exhibited sensitivity to the activator CaCl₂, with only minimal changes observed in the MPTP group (Figure 8A). In response to the inhibitor cyclosporine A, only Group 3 demonstrated sensitivity, while no other group showed a significant response (Figure 8B).

**Figure 9:**
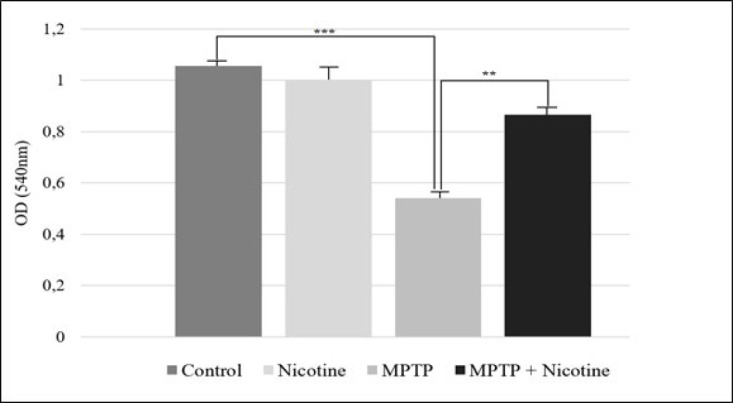
The mitochondrial swelling in substantia nigra cells

This study also examined mitochondrial swelling as a downstream event following the opening of the mitochondrial permeability transition pore. Our results indicate that MPTP treatment significantly increased mitochondrial swelling, as evidenced by reduced absorbance (*P*≤0.0001). In contrast, nicotine effectively prevented mitochondrial swelling in isolated mitochondria, showing a highly significant difference compared to the MPTP group (*P*≤0.001), as shown in Figure 9.

### PI3K-AKT-mTOR pathway

**Figure 10:**
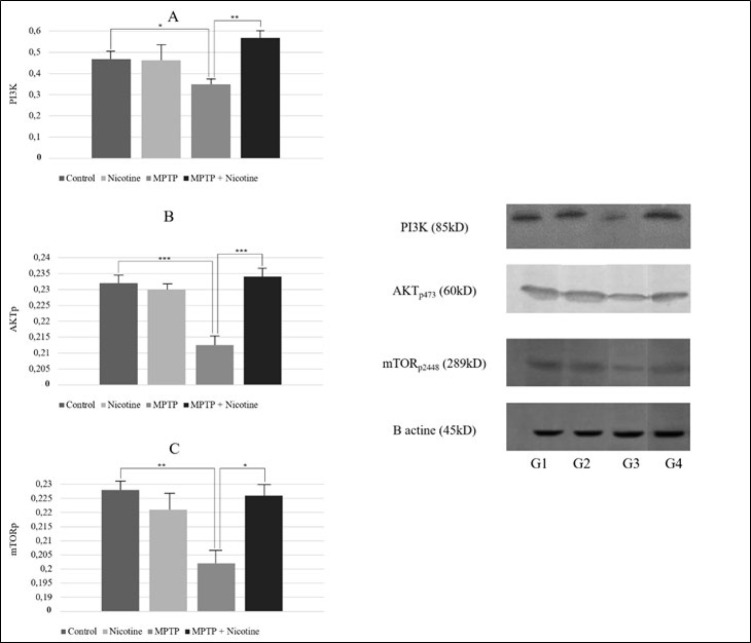
Mean values and representative Western blots of PI3K, p-AKT(Serine 473) and p-mTOR (Serine 2448) in the SN cells

It is known that various internal and external processes regulate the course of energy metabolism. Among these processes, the PI3K-AKT-mTOR signaling pathway plays a central role. Taking this into account, the quantitative changes of some components of this pathway in the cells of the substantia nigra of the brain of the research groups were studied in further tests. The obtained data are presented in Figure 10. As shown in Figure 10, the PI3K-AKT-mTOR signaling pathway is inhibited in the MPTP group. Nicotine activates the pathway by increasing the phosphorylation of AKT (*P*≤0.0001), which subsequently enhances the activation of mTOR (*P*≤0.01). 

## Discussion

PD is a progressive, long-term neurodegenerative disorder primarily characterized by the massive loss of dopaminergic neurons in the substantia nigra pars compacta and the formation of protein aggregates, known as Lewy bodies composed of synuclein (41). PD is believed to result from a complex interplay of aging, genetic predispositions, and environmental factors. These interactions lead to mechanisms that disrupt dopaminergic neuron function, causing oxidative stress, mitochondrial dysfunction, inflammation, and genetic mutations that impair the ubiquitin-proteasome system, ultimately resulting in neurodegenerative changes (42-43). Currently, 120–150 individuals per 100,000 are affected by PD, and given the increasing prevalence rates, the number of patients is expected to rise significantly by 2050. Consequently, there is a growing focus on finding interventions to mitigate neurodegenerative processes.

Nicotine, the primary alkaloid found in tobacco, constitutes approximately 0.6–3.0% of its dry weight (44). Remarkably, epidemiological investigations have indicated that individuals who smoke cigarettes exhibit a reduced mortality rate from PD when compared to non-smokers, thereby suggesting a potential influence of nicotine on the course of this debilitating disorder (45-46). Furthermore, the neuroprotective properties of nicotine have been substantiated through diverse in vivo studies (47-49). These investigations not only underscore the advantageous impact of nicotine in animal models of Parkinsonism but also elucidate various signaling pathways implicated in this phenomenon. Notably, these pathways include the downregulation of SIRT-6, the inhibition of PARP-1 and caspase-3 cleavage, as well as the modulation of the p-JNK/JNK and p-ERK/ERK ratios. It is conceivable that the neuroprotective efficacy of nicotine extends beyond these pathways, encompassing other cellular regulatory mechanisms. Of particular interest is the inquiry into whether nicotine’s action is associated with a reduction in oxidative stress, a key pathological process in the context of PD. Intriguingly, some studies have suggested that nicotine may paradoxically elevate oxidative stress levels. Our previous study showed that nicotine administration improved the locomotor function of MPTP-treated mice, elevated dopamine levels, and decreased markers of oxidative stress. Nicotine also increased the activity of anti-oxidant enzymes in the cells of the substantia nigra (12). In this study, we investigated the effects of chronic, per oral nicotine administration on the energetical metabolism of substantia nigra (SN) cells in an MPTP-induced PD model. Our results indicate that nicotine exerts a neuroprotective role, particularly through modulation of key mitochondrial and metabolic processes. This suggests potential therapeutic pathways for mitigating energy deficits characteristic of PD. 

MPTP administration led to a marked inhibition of glycolysis, the TCA cycle, and oxidative phosphorylation, indicating severe mitochondrial dysfunction. This observation aligns with existing literature that implicates mitochondrial impairment in PD pathogenesis, particularly through inhibition of Complex I of the electron transport chain (ETC) (50). In our study, the activities of key enzymes involved in glycolysis and the TCA cycle, such as aldolase, pyruvate dehydrogenase, aconitase, α-ketoglutarate dehydrogenase, and malate dehydrogenase, were significantly disrupted by MPTP, resulting in reduced ATP production and an energy deficit. Unlike other enzymes affected by the neurotoxin, hexokinase and fumarase exhibited different responses: hexokinase activity increased, while fumarase activity remained unchanged. The increase in hexokinase activity can be explained as a compensatory mechanism, where the cell attempts to break down more glucose to compensate for the lack of ATP (51). In contrast, fumarase’s resistance to oxidative stress can be attributed to its structural features, particularly the absence of sulfhydryl bonds, which protects it from the damaging effects of the neurotoxin (52). Nicotine administration, however, restored the activity of these enzymes and normalized ATP production, suggesting that nicotine modulates mitochondrial function to counteract MPTP-induced damage. The restoration of TCA cycle enzyme activity points toward nicotine’s ability to enhance mitochondrial biogenesis or stability, possibly through its effects on mitochondrial dynamics. 

The alterations in creatine kinase (CK) activity further underscore the metabolic dysregulation caused by MPTP. The nearly threefold decrease in mitochondrial CK activity following MPTP administration reflects impaired energy buffering in the mitochondria. This disruption compromises the ability of cells to rapidly mobilize ATP stores in response to energy demands, a critical feature in neurons that rely on efficient energy transfer for synaptic function (53-54). Nicotine’s ability to normalize CK activity in both mitochondrial and cytosolic compartments suggests that it plays a crucial role in stabilizing energy metabolism at multiple levels. By restoring the CK shuttle system, nicotine ensures that neurons can efficiently meet energy demands, especially under conditions of mitochondrial stress. This normalization may also contribute to nicotine’s protective effects on dopaminergic neurons, where energy deficits are a key driver of neurodegeneration.

One of the most intriguing findings of this study is nicotine’s differential regulation of electron transport chain (ETC) complexes. As expected, the inhibition of Complex I in the MPTP group aligns with MPTP’s known neurotoxic mechanism, which directly impairs this complex. Interestingly, nicotine not only restored Complex I activity but also modulated Complex II, which had been paradoxically increased in the MPTP group. The elevated activity of Complex II likely represents a compensatory response to the inhibition of Complex I, as cells attempt to maintain ATP synthesis despite the disruption. This compensatory mechanism may involve reverse electron transport (RET), a process in which electrons flow backward from Complex II to Complex I (55). In the case of MPTP-induced Complex I inhibition, RET allows electrons to be pushed from Complex II upstream to Complex I, resulting in heightened Complex II activity. However, while RET can temporarily support ATP production, it has also been associated with increased reactive oxygen species (ROS) production (56). On the other hand, given that Complex I inhibition is sustained, it is possible that the increase in the activity of the second complex is caused by a compensatory mechanism and serves to enhance the transport of electrons not to the first but to the third complex. Nicotine’s ability to normalize both Complex I and II activities suggests that it may mitigate the adverse effects of MPTP, supporting more efficient and balanced mitochondrial function in the context of ETC dysfunction. Additionally, the activities of Complexes III and IV, which were also suppressed by MPTP, were significantly restored by nicotine treatment. This broader normalization of ETC function suggests that nicotine plays a pivotal role in optimizing electron flow across the entire chain, potentially reducing excessive ROS production. Complex II’s dual role in both the TCA cycle and the ETC makes it a critical node for regulating cellular metabolism, and its dysregulation is known to contribute to ROS generation (57). Nicotine’s ability to restore Complex I and other complex activities may be attributed to its indirect anti-oxidant effects, as previous studies have demonstrated that nicotine enhances the activity of anti-oxidant enzymes such as superoxide dismutase (SOD) and catalase, preventing oxidative damage to ETC components (12). The observed increase in ATP synthase (Complex V) activity further supports the hypothesis that nicotine enhances ATP production efficiency, contributing to energy homeostasis under neurodegenerative conditions.

The PI3K-AKT-mTOR pathway is a central regulator of cellular metabolism, growth, and survival (58). Our results demonstrate that MPTP significantly inhibited this pathway, as reflected by reduced phosphorylation of AKT and mTOR. This inhibition likely exacerbates energy deficits by impairing glucose metabolism and reducing the cell’s ability to respond to metabolic stress. Nicotine, however, reactivated the pathway by increasing the phosphorylation of AKT, which in turn enhanced mTOR activation (26). The reactivation of the PI3K-AKT-mTOR pathway by nicotine could have far-reaching effects on cellular metabolism. mTOR signaling is known to regulate glycolysis by modulating the activity of hexokinase and other glycolytic enzymes (59). Thus, nicotine’s ability to activate mTOR may explain the observed normalization of glycolytic enzyme activity in the nicotine-treated group. Additionally, mTOR plays a protective role in preventing mitochondrial permeability transition pore (mPTP) opening, a process that leads to mitochondrial swelling and cell death (29). By inhibiting mPTP opening, nicotine may prevent the loss of mitochondrial membrane potential, thereby preserving mitochondrial function and preventing apoptosis in SN cells. In particular, there was a decrease in sensitivity to calcium chloride, a known activator of the mitochondrial permeability transition pore, in the MPTP group, along with an enhanced response to the mPTP blocker, cyclosporine A. This observation suggests that the mitochondrial pore in this group is already open due to the underlying pathological process. Consequently, these mitochondria exhibit swelling, which serves as a characteristic marker of mitochondrial dysfunction (60).

Our study provides compelling evidence that nicotine exerts neuroprotective effects on SN neurons in an MPTP-induced PD model by modulating multiple aspects of energy metabolism. Nicotine’s ability to restore glycolytic, TCA cycle, and ETC enzyme activity, normalize CK function, and activate the PI3K-AKT-mTOR pathway suggests that it functions as a potent regulator of neuronal energetics. These findings open up new avenues for investigating nicotine and related compounds as potential therapeutics for PD, particularly in targeting the metabolic disturbances that underlie dopaminergic neurodegeneration. Further research is needed to elucidate the precise molecular mechanisms by which nicotine exerts these effects and to explore its therapeutic potential in clinical settings.

Glucose undergoes metabolism via glycolysis, resulting in the production of pyruvate. Pyruvate is then converted into acetyl-CoA by the enzyme pyruvate dehydrogenase and enters the tricarboxylic acid (TCA) cycle. During the TCA cycle, reduced cofactors are generated, which transfer electrons to the mitochondrial electron transport chain, ultimately producing ATP. Mitochondrial creatine kinase catalyzes the transfer of a phosphate group from ATP to creatine, forming phosphocreatine (P-Creatine) and ADP. In the cytoplasm, cytosolic creatine kinase reverses this reaction, converting phosphocreatine back to creatine while regenerating ATP from ADP. This phosphocreatine system serves as a rapid energy buffer and shuttle between mitochondria and cytosolic ATP-consuming processes. Phosphoinositide 3-kinase (PI3K) activates protein kinase B (AKT) via phosphorylation. Activated AKT subsequently phosphorylates and activates the mechanistic target of rapamycin (mTOR). mTOR signaling enhances glycolysis and promotes closure of the mPTP. Under pathological conditions, the mPTP remains open, leading to mitochondrial swelling, release of pro-apoptotic factors, and activation of apoptotic signaling pathways.

The reported results represent the mean or average values obtained from 10 repeated trials. Note: Data are mean ± SEM; *P≤0.01; ** *P*≤0.001; ***P≤0.0001

The reported results represent the mean or average values obtained from 10 repeated trials. Note: Data are mean ± SEM; * *P*≤0.01; ** *P*≤0.001; ***P≤0.0001.

The reported results represent the mean or average values obtained from 10 repeated trials. Note: Data are mean ± SEM; * *P*≤0.01; ** *P*≤0.001; *** *P*≤0.0001

The reported results represent the mean or average values obtained from 10 repeated trials. Note: Data are mean ± SEM; * P≤0.01; ** *P*≤0.001; *** *P*≤0.0001

The reported results represent the mean or average values obtained from 10 repeated trials. Note: Data are mean ± SEM; * *P*≤0.01; ** *P*≤0.001; *** *P*≤0.0001.

The reported results represent the mean or average values obtained from 10 repeated trials

The reported results represent the mean or average values obtained from 10 repeated trials. Note: Data are mean ± SEM; * *P*≤0.01; ** *P*≤0.001; *** *P*≤0.0001

The data shown are representative of three independent experiments. β-Actin was also visualized by Western blotting to conﬁrm the equal loading of the fractions. Quantification of PI3K blots is shown in A, of AKTp in B, and of mTORp in C. Note: Data are mean ± SEM; * *P*≤0.01; ** *P*≤0.001; *** *P*≤0.0001

## Conclusion

Nicotine restores mitochondrial function and ATP levels in MPTP-induced PD models, likely through activation of the PI3K-AKT-mTOR pathway, suggesting its potential as a neuroprotective agent.

## Conflicts of Interest

The authors declare no conflicts of interest regarding the publication of this paper.

## Declaration

During the preparation of this work, the author(s) used ChatGPT to improve language and grammar. After using this tool/ service, the author(s) reviewed and edited the content as needed and take(s) full responsibility for the publication’s content.

## Data Avaiiability Statement

Data sharing does not apply to this article as no datasets were generated or analyzed during the current study.
